# Histone deacetylation of memory T lymphocytes by *You-Gui-Wan* alleviates allergen-induced eosinophilic airway inflammation in asthma

**DOI:** 10.1186/s13020-015-0038-9

**Published:** 2015-05-13

**Authors:** Hong Ping Zhang, Juan Juan Fu, Tao Fan, Wen Bin Zhang, Zeng Li Wang, Lei Wang, Gang Wang

**Affiliations:** Pneumology Group, Department of Integrated Traditional Chinese and Western Medicine, State Key Laboratory of Biotherapy of China, West China Hospital, Sichuan University, Chengdu, 610041 PR China; Pneumology Group, Department of Integrated Traditional Chinese and Western Medicine, West China Hospital, Sichuan University, Chengdu, 610041 PR China; Department of Respiratory Medicine, Chongqing Traditional Chinese Medicine Hospital, Chongqing, 400011 PR China; Department of Respiratory and Critical Care Medicine, West China Hospital, Sichuan University, Chengdu, 610041 PR China

## Abstract

**Background:**

You-Gui pills (*You-Gui-Wan*; YGW) can promote T lymphocyte proliferation and differentiation, and restore Th1/Th2 balance in the treatment of asthma, but their mechanism of action is not fully known. This study aims to explore whether YGW can induce histone deacetylation or acetylation in memory T lymphocytes (Tm) for improvement of airway inflammation in asthma.

**Methods:**

CD4^+^CD45RB^low^ cells, as Tm, were obtained by magnetic-activated cell sorting and flow cytometry from the spleens of BALB/c mice with ovalbumin (OVA)-induced asthma. Tm were cocultured with hydrocortisone (CORT; 1000 nM), serum containing low (0.225 g/kg), moderate (0.9 g/kg), or high (3.6 g/kg) doses of YGW, or medium only, and then adoptively transferred into naïve mice (*n* = 5 per group). Recipient mice were challenged with aerosolized OVA. The levels of IL-4, IL-5, IL-13, and IFN-γ in culture supernatants and bronchoalveolar lavage fluid (BALF) from the OVA-challenged mice were measured by ELISA. Histone deacetylase (HDAC) and histone acetyltransferase (HAT) activities and protein expressions of T-bet, GATA-3, and HDAC1–11 in lung tissue were measured by western blotting analyses. The alveolar eosinophilic inflammation index (AEII) was evaluated in the lungs of adoptive transfer recipient mice.

**Results:**

YGW reduced inflammation and eosinophil infiltration into the lung tissues as evidenced by histology, with similar effects to those of CORT. High-, moderate-, and low-YGW increased HDAC (*P* < 0.0001, *P =* 0.0009 and *P =* 0.0253 respectively) and decreased HAT (*P* = 0.0001, *P =* 0.0000 and *P =* 0.0039, respectively) activities in dose-dependent manners in the lung tissues of adoptive transfer recipient mice. Increased histone deacetylation of Tm by YGW reduced the AEII by reducing GATA-3 (*P* = 0.014),IL-4 (*P =* 0.0004), IL-5 (*P* = 0.0067), and IL-13 (*P* = 0.0002), and inducing IFN-γ release (*P* = 0.0375). Moreover, YGW reduced inflammatory cytokines such as IL-4, IL-5, and IL-13 by upregulating the activities of HDAC7 (*P* = 0.003)/10 (*P* = 0.003), HDAC11 (*P* < 0.0001), and HDAC9–11 (*P* < 0.0001, *P* < 0.0001 and *P* < 0.0001, respectively), respectively, and increased IFN-γ release by increasing HDAC9 (*P* < 0.0001).

**Conclusions:**

Histone deacetylation of Tm was observed during alleviation of allergen-induced eosinophilic airway inflammation in asthma by YGW.

## Introduction

Asthma is an inflammatory disease of the airways that involves multiple inflammatory cells (T lymphocytes and eosinophils) and inflammatory mediators [1,2]. Allergic asthma is considered to be a Th2-driven inflammatory disease [3]. Therefore, drugs that can suppress Th2 cytokine production are potentially therapeutic [4].

Histone acetylation is an epigenetic modification that maintains pre-established patterns of cytokine memory [5]. Zhang *et al.* [6] identified T cells with a memory-like phenotype during fetal development, which displayed a large variety of inflammatory effector functions associated with CD4^+^ T helper (Th) cells at birth. Importantly for immune memory, the histone modification profile at a given gene locus could be inherited through mitosis [7,8]. Histone acetyltransferases (HATs) and histone deacetylases (HDACs) regulate chromatin structure and affect inflammatory gene expressions [9]. The extents of acetylation of core histones might reflect the balance between the opposing activities of HATs and HDACs [10]. Acetylation of histone tails is involved in the activation of gene transcription [11] and enhances chromatin accessibility [10]. Su *et al.* [12] found that endogenous HDAC activity was involved in maintaining the balance of pre-established Th1-like and Th2-like responses, thereby inhibiting excessive Th2 immunity.

Although corticosteroids and β_2_-agonists can improve asthma symptoms, there are known side effects, such as disturbance of adrenal function and generalized immune suppression, particularly in children [13]. Some Chinese medicines promote T lymphocyte proliferation and transformation, and adjust imbalances in Th1 and Th2 responses [14]. You-Gui pills (*You-Gui-Wan*; YGW) have efficacy for the treatment and prevention of asthma [15] by enhancing the immune system’s ability [16]. However, the mechanisms underlying the effects of YGW in asthma treatment are still unknown.

This study aims to explore whether YGW could induce histone deacetylation or acetylation in memory T lymphocytes (Tm) for improvement of airway inflammation in asthma.

## Methods

### Mice

**Figure 1:**
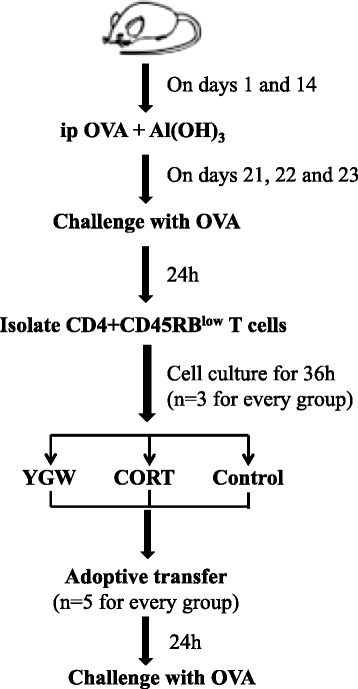
Flow chart outlining the study protocol for OVA sensitization and challenge and/or adoptive transfer. i.p., intraperitoneal injection.

Male BALB/c mice (6–8weeks of age and free of murine-specific pathogens) were purchased from the Laboratory Animal Center of West China School of Medicine (China). The mice were housed throughout the experiments in a laminar flow cabinet. The animal study protocols were approved by the Institutional Animal Care Committee of West China Hospital, Sichuan University, China (Supplementary file 01). The flow chart for this study protocol was shown in Figure 1.

### Serum preparation containing YGW

YGW capsules (batch number: 961015) were purchased from YinTao Pharmaceutics Ltd. (China). YGW capsules (4,500.00 mg/capsule) were dissolved in saline (473.68 mg/mL) and stored at −20°C before administration to mice. Thirty-six mice were randomly divided into YGW (*n* = 12) and sham control (*n* = 24) groups, and then gavaged (3.6 g/kg body weight) continuously for 5 days with YGW or saline (200 μL), respectively. The mice were anesthetized at 2 h after the last gavage, and blood was withdrawn and centrifuged (H2050R; Xiangyi, China) at 12,000 rpm (10,005 × *g*) for 4 min at 4°C. The supernatant, designated the decoction serum, was inactivated for 30 min at 56°C and stored at −80°C until use.

### Allergen sensitization and challenge

Mice were sensitized and challenged with ovalbumin (OVA) (Sigma-Aldrich, USA) as previously described [17]. Briefly, mice were sensitized on days 1 and 14 by intraperitoneal injection of 20mgof OVA emulsified in 1 mg of aluminum hydroxide (Longsheng Chemical Co. Ltd., China) in a total volume of 200 μL. On days 21, 22, and 23 after the initial sensitization, the mice were challenged for 30 min with an aerosol of 30 μg/mL OVA in PBS by placing the mice in a 20 × 30 × 50-cm plexiglass chamber connected to an ultrasonic nebulizer (Berry Co. Ltd., Germany) that generated an aerosol mist with a pumping volume of 6 L/min.

### Isolation and purification of CD4^+^CD45RB^low^ T cells

Mice were euthanized at 24 h after the last OVA challenge, and their spleens were removed aseptically. Lymphocytes were obtained from the spleens using an EZ-Sep™ Mouse1 × Lymphocyte Separation Kit (DakeweiBiotech Company, China). CD4^+^ T lymphocytes were negatively selected by magnetic-activated cell sorting (Dynal® Mouse CD4 Negative Isolation Kit, Invitrogen Dynal, USA) according to the manufacturer’s instructions.

**Figure 2:**
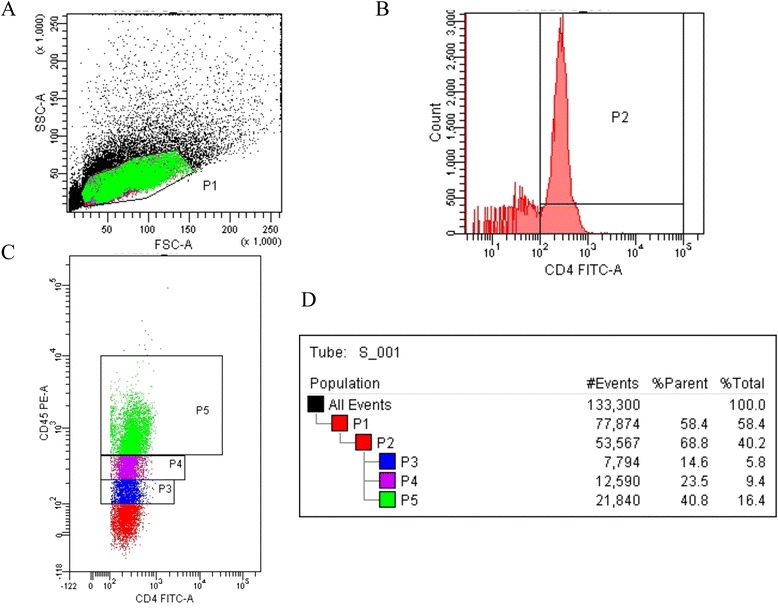
Flow cytometry to isolate CD4^+^CD45RB^low^ cells. **(A)** Lymphocytes (P1). **(B)** CD4^+^ T lymphocytes (P2). **(C)** CD4^+^CD45RB^low^ cells (P3) and CD4^+^CD45RB^high^ cells (P5). **(D)** Proportion of cells in each group.

CD4^+^CD45RB^low^ T cells were obtained by flow cytometry (FC 500 Series Flow Cytometry System; Beckman Coulter, USA) as previously described [18]. Briefly, CD4^+^ T cells were incubated with FITC-conjugated anti-CD4 (Clone GK1.5; BD Pharmingen™, USA) and PE-conjugated anti-CD45RB (Clone 16A; BD Pharmingen™, USA) antibodies in PBS for 30 min at 4°C, and washed twice in PBS. The cells were then sorted by flow cytometry using a FACS Vantage SE Cell Sorter (Beckman Coulter Company, USA) (Figure 2). The purity of CD4^+^CD45RB^low^ cells was >95%.

### Cell culture, interventions, and cytokine analysis

Isolated CD4^+^CD45RB^low^cells were cultured in RPMI 1640 medium (Gibco, USA) at 1.5× 10^5^ cells/mL in 24-well round-bottom plates (BD,USA) in triplicate. The cells were treated with 5 μg/mL concanavalin A (BD, USA), 100U/mL of penicillin and streptomycin (Shandong Lukang Pharmaceutical Company, China), and various interventions at 37°C in a 5% CO_2_ incubator for 36 h. The interventions were as follows: (1) high dose of YGW (3.6 g/kg; H-YGW), given as 200 μLof YGW serum, comprising 40 times the equivalent clinical dose given to adults; (2) moderate dose of YGW (0.9 g/kg; M-YGW), given as 50 μL of YGW serum and 150 μL of blank serum; (3) low dose of YGW (0.225 g/kg; L-YGW), given as 12.5 μL of YGW serum and 187.5 μL of blank serum; (4) hydrocortisone (CORT), given as hydrocortisone (1000nM) (Sigma, USA) and 200 μL of blank serum; and (5) control group (OVA), given as 200 μL of blank serum without any drug intervention. After culture, the supernatants were kept at −80°C until analysis by ELISA (Senxiong Biotech, China) for IL-4, IL-5, IL-13, and IFN-γ, and the CD4^+^CD45RB^low^ cells were used for adoptive transfer to naïve mice.

### Adoptive transfer, challenge, bronchoalveolar lavage, and cytokine analysis

The CD4^+^CD45RB^low^ cells in each intervention were resuspended in 250 μL of PBS, divided into five equivalent parts, and adoptively transferred to five naïve mice (approximately 0.9 × 10^5^ cells/mouse). The adoptive transfer was performed as described in our previous report [19]. Briefly, anesthetized and tracheostomized naïve mice were injected with 50 μL of CD4^+^CD45RB^low^ cell suspension using a syringe. At 24 h after adoptive transfer, the mice were challenged for 30 min with OVA.

Bronchoalveolar lavage was performed at 24 h after the last challenge. The left lungs were lavaged three times with 1 mL of sterile normal saline, as previously described [20]. Briefly, the mice were euthanized with sodium pentobarbitone (100 mg/kg body weight, administered intraperitoneally). The chest cavity was exposed for expansion, and the trachea was cannulated with a 20-gauge catheter that was secured with a ligature. Lavage of the left lung was performed using a 1-mL aliquot of saline. A total of three lavages were performed after tying off the right lung at the mainstem bronchus. In all mice, the recovery of the lung lavage fluid was 80% or greater. The recovered fluid was centrifuged at 1,200 rpm (273 × *g*) and 4°C for 5 min. The supernatants of the bronchoalveolar lavage fluid (BALF) were kept at −80°C until analysis by ELISA for IL-4, IL-5, IL-13, and IFN-γ (Senxiong Biotech, China).

### Lung tissue pathology and alveolar eosinophilic inflammation index (AEII)

The right lower lung tissues around the main bronchus were cut, inflated with 4% formaldehyde solution, and embedded in paraffin. Cryostat sections (4 μm) of the tissues were cut and air-dried. Morphological observations were performed after sections were stained with hematoxylin and eosin (HE), and asthma-like inflammation features were assessed in each section under 400× magnification. The AEII was assessed as previously described [19]. Briefly, the eosinophilic inflammation intensity in the alveolar area was calculated as the number of eosinophils per alveolar area. Three sections were arbitrarily selected from each group of mice and three alveolar regions in each section were analyzed at high magnification, while trying to avoid blood vessels and bronchioles. The eosinophils in each zone were counted, and the area of each alveolar region was determined using Image-Pro Plus 4.5 software (Media Cybernetics, USA). These data were used to calculate the average density of eosinophils in each group, for measurement of the eosinophilic inflammation intensity. The AEII was calculated as the inflammation intensity ratio of each group versus naïve mice.

### HDAC and HAT activities

The HDAC and HAT activities in the right upper lung tissues were measured using colorimetric activity assay kits (Colorimetric HDAC Activity Assay Kit [Catalog #K331-100); HAT Activity Colorimetric Assay Kit [Catalog #K332-100); BioVision Inc.,USA], in accordance with the manufacturer’s protocols. Briefly, an HDAC colorimetric substrate was incubated with 80 μg of total nuclear extracts from lung tissues for 60 min at 37°C. Following the incubation, lysine-developer solution was added to generate a measurable chromophore that was analyzed by the absorbance at 405 nm using an ELISA microplate reader (Bio-Rad Laboratories, USA). HeLa cells nuclear extracts (4 μg) were used as a positive control. An HDAC inhibitor, trichostatin A, was used to demonstrate the specificity of the deacetylation activities. For the HAT activity assay, an active nuclear extract as a positive control and acetyl-CoA as a cofactor were utilized. Total nuclear extracts (50 μg) were incubated with acetyl-CoA at 37°C for 120 min. Acetylation of the peptide substrate by active HAT released the free form of CoA, which then served as an essential coenzyme for producing NADH. The produced NADH was detected spectrophotometrically upon reacting with a soluble tetrazolium dye. The activity was analyzed by the absorbance at 440 nm using the above-described ELISA plate reader.

### Western blot analysis

The right upper lung tissues were homogenized in the presence of protease inhibitors, and the protein concentrations were determined using NE-PER® nuclear and cytoplasmic extraction reagents (Pierce Biotechnology, USA). The total proteins (50 μg) were loaded onto SDS-PAGE gels. After electrophoresis at 120 V for 90 min, the separated proteins were transferred to polyvinylidene difluoride membranes using a wet transfer method (250 mA for 90 min) [21]. Nonspecific sites were blocked with 5% nonfat dry milk in Tris-buffered saline containing 0.1% Tween 20 for 1 h, and the membranes were then incubated overnight at 4°C with anti-GATA-3, anti-T-bet (Santa Cruz Biotechnology Inc., USA), or anti-HDAC1–11 (HDAC Family Antibody Set; BioVision, USA) primary antibodies. Horseradish peroxidase-conjugated anti-rabbit IgG was used to detect binding of the primary antibodies. The membranes were stripped and reprobed with an anti-actin primary antibody (Sigma-Aldrich, USA) to verify equal protein loading in each lane. The binding of the specific antibodies was visualized by exposure to photographic film. The densities of the stained bands for T-bet, GATA-3, and HDAC1–11 relative to the staining band for β-actin were quantified with Quantity ONE densitometry software (PDI Imageware Systems, USA).

### Statistical analysis

All data were presented as mean ± SD. Differences among data were analyzed by one-way analysis of variance (ANOVA). For comparisons of data that were not normally distributed, a Mann–Whitney *U*-test was performed. Student-Newman-Keuls (SNK) test was used for multiple group comparisons. The dose-dependent manner was visually determined. Associations were investigated by Spearman correlation analysis. A double-sided *P* value of < 0.05 was considered statistically significant. All statistical analyses were performed using Stata version 11.0 (Stata Corp LP, USA).

## Results

### Cytokines in cell culture supernatants and BALF

Tm were cocultured with CORT, serum containing low, moderate, or high doses of YGW, or medium only. The levels of IL-4, IL-5, IL-13, and IFN-γ in culture supernatants and BALF from OVA-challenged mice were measured by ELISA (Table 1). CORT significantly reduced the IL-4 (*P* = 0.0161), IL-5 (*P* = 0.0059), and IL-13 (*P* = 0.0005) levels. H-YGW, M-YGW and L-YGW significantly reduced the IL-4 (*P* = 0.0002, *P* = 0.0008 and *P* = 0.0015, respectively) and IL-5 (*P* = 0.0004, *P* = 0.0008 and *P* = 0.0015, respectively) levels. Compared with CORT, the IL-4 level in H-YGW cultures was decreased to a greater extent (*P* = 0.0167). There were dose-dependent responses to YGW in reducing the IL-4, IL-5, and IL-13 levels. The IFN-γ level in the H-YGW cultures was increased compared with the OVA cultures (*P* = 0.0164).

**Table 1:** **IL-4, IL-5, IL-13, and IFN-γ levels (pg/mL) in cell culture supernatants and BALF**

**Cytokines**	***You-Gui-Wan***			**CORT**	**OVA**
	**H-YGW**	**M-YGW**	**L-YGW**		
Cell culture	n = 3	n = 3	n = 3	n = 3	n = 3
IL-4	9.9 ± 2.4^**†^	17.4 ± 1.9^**^	17.5 ± 2.5^**^	19.4 ± 4.6^*^	28.5 ± 1.7
IL-5	14.2 ± 2.0^**^	17.8 ± 9.3	21.2 ± 6.4	15.0 ± 4.4^**^	26.6 ± 1.2
IL-13	25.4 ± 16.2^*^	35.3 ± 19.4	40.2 ± 18.9	15.9 ± 2.8^*^	45.5 ± 5.2
IFN-γ	3.4 ± 0.2^*^	2.9 ± 0.5	2.8 ± 0.2	3.1 ± 0.2	2.9 ± 0.3
BALF	n = 5	n = 5	n = 5	n = 5	n = 5
IL-4	18.6 ± 6.8^**^	27.7 ± 11.0	21.3 ± 5.0^**^	24.2 ± 3.4^**^	36.4 ± 3.4
IL-5	12.4 ± 5.2^**^	18.7 ± 5.3	23.3 ± 5.4	14.1 ± 2.5^**^	19.9 ± 1.2
IL-13	9.1 ± 1.5^**^	17.5 ± 2.8^**^	32.8 ± 5.4	9.9 ± 7.5^**^	27.5 ± 6.8
IFN-γ	2.9 ± 0.2^*^	2.7 ± 0.4	2.4 ± 0.2	2.4 ± 0.2	2.6 ± 0.3

^*^ *P* <0.05, ^**^ *P* < 0.01, compared with OVA. ^†^ *P* <0.05, compared with CORT.

The IL-4, IL-5, IL-13, and IFN-γ levels in BALF after adoptive transfer are shown in Table 1. CORT significantly reduced the IL-4 (*P* = 0.0002), IL-5 (*P* = 0.0008), and IL-13 (*P* = 0.0023) levels, H-YGW significantly reduced the IL-4 (*P* = 0.0004), IL-5 (*P* = 0.0067) and IL-13 (*P* = 0.0002) levels, but there were no differences in the levels of these cytokines between YGW and CORT (all *P* > 0.05). The levels of IL-4, IL-5, and IL-13 showed dose-dependent responses to YGW. The IFN-γ level in the H-YGW group was much higher than that in the OVA group (*P* = 0.0375).

### HDAC and HAT activities

**Figure 3:**
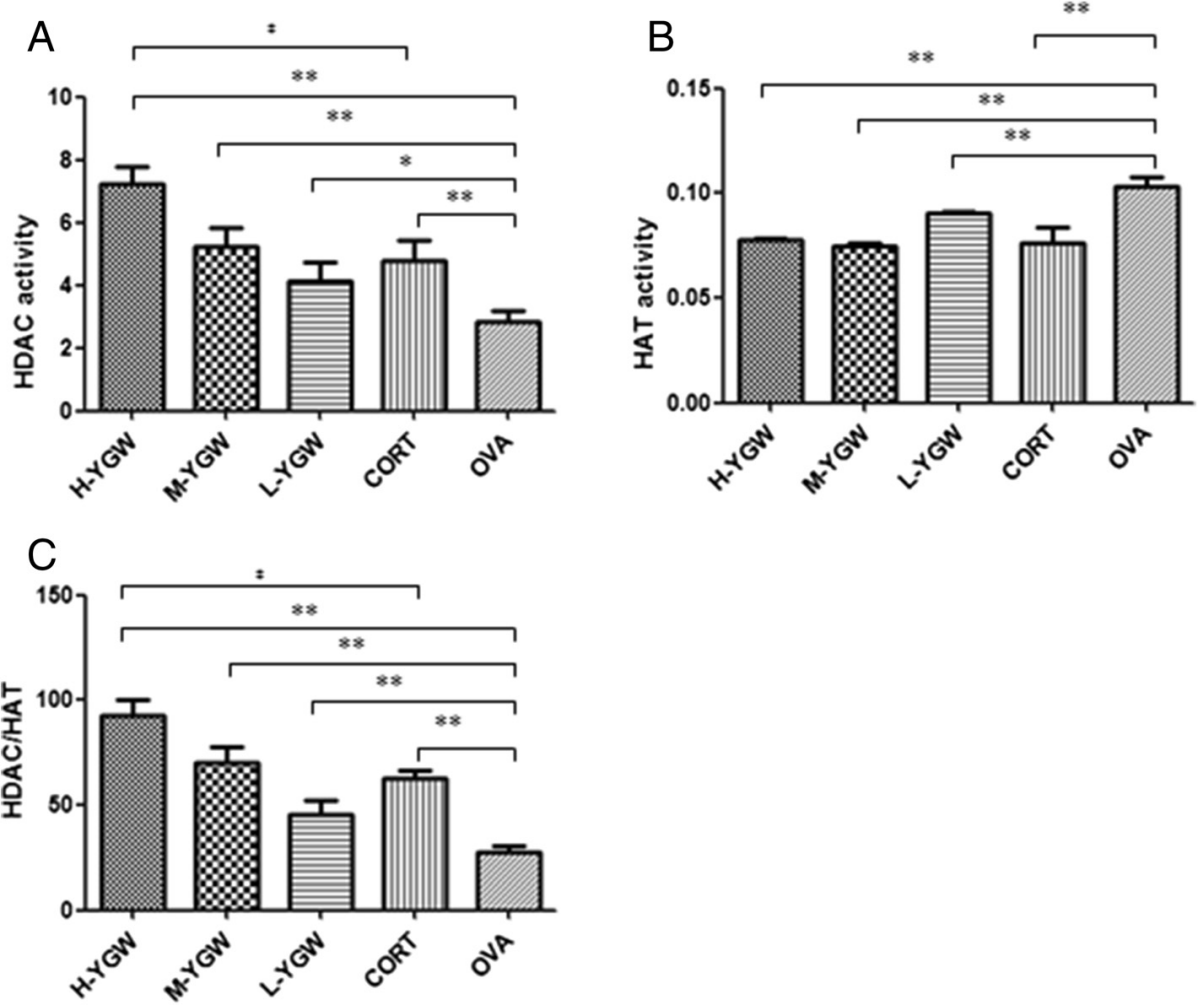
Total HDAC activity (μM/μg) **(A)**, HAT activity (μM/μg) **(B)**, and HDAC/HAT ratio **(C)** in the lung tissues of mice after adoptive transfer. *P <0.05, **P<0.01 compared with OVA. ^‡^P <0.01, compared with CORT. Student-Newman-Keuls (SNK) test was used for multiple group comparisons.

The HDAC and HAT activities in the lung tissues of mice after adoptive transfer are shown in Figure 3. The HDAC activities and ratios of HDAC to HAT activity (HDAC/HAT) in the H-YGW (*P* = 0.0004 and *P =* 0.0002 respectively), M-YGW (*P* = 0.0009 and *P =* 0.0001 respectively), L-YGW (*P* = 0.0253 and *P =* 0.0053 respectively) and CORT groups (*P* = 0.0037 and *P =* 0.0000 respectively) were significantly higher than those in the OVA group. By contrast, the HAT activities in the H-YGW (*P =* 0.0067), M-YGW (*P =* 0.0000), L-YGW (*P =* 0.0039) and CORT (*P =* 0.0015) groups were significantly decreased compared with the OVA group. The HDAC activity, HAT activity, and HDAC/HAT ratio showed dose-dependent responses to YGW.

### Expressions of GATA-3, T-bet, and HDAC1–11 proteins in lung tissue

**Figure 4:**
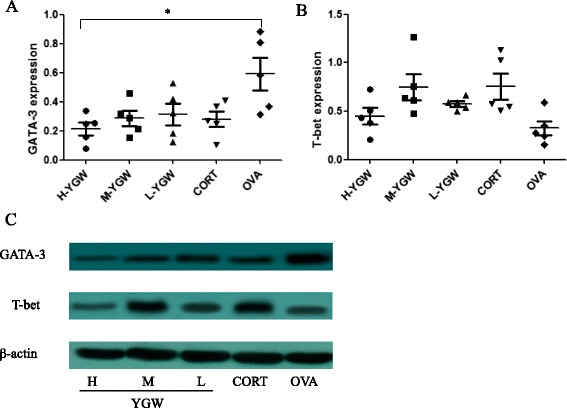
GATA-3 **(A)** and T-bet **(B)** protein expressions, normalized by β-actin expression **(C)**, in the lung tissues of mice after adoptive transfer. ^*^ *P* <0.05, compared with OVA. Student-Newman-Keuls (SNK) test was used for multiple group comparisons.

The expressions of GATA-3 and T-bet proteins in the lung tissues of mice after adoptive transfer are shown in Figure 4. The GATA-3 expression in the YGW group was significantly lower than that in the OVA group (*P* = 0.014), but the observed downregulation in the CORT group did not reach statistical significance (*P* = 0.062). There were no significant differences in T-bet expression between the YGW, CORT, and OVA groups.

**Figure 5:**
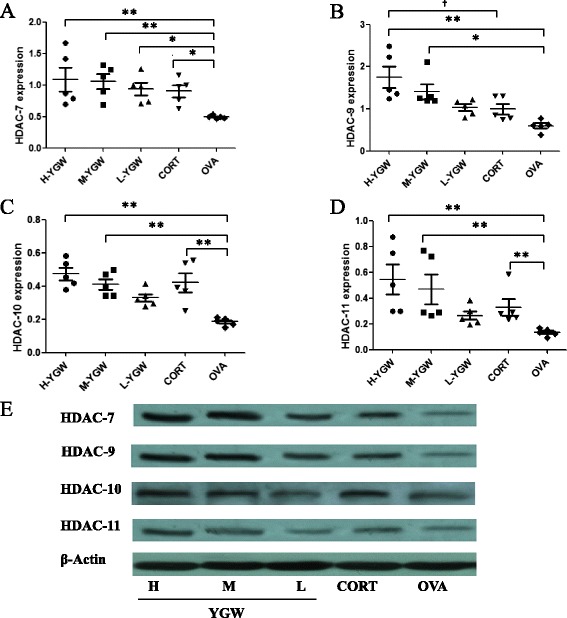
HDAC7 **(A)**, HDAC9 **(B)**, HDAC10 **(C)**, andHDAC11 **(D)** protein expressions, normalized by β-actin expression **(E)**, in the lung tissues of mice after adoptive transfer, determined by western blotting. ^*^ *P* <0.05, ^**^ *P* < 0.01, compared with OVA.^†^ *P* <0.05, compared with CORT. Student-Newman-Keuls (SNK) test was used for multiple group comparisons.

The expressions of HDAC7, HDAC9, HDAC10, and HDAC11 in the lung tissues of mice after adoptive transfer are shown in Figure 5. The expressions of HDAC7, HDAC10, and HDAC11 in the H-YGW (*P* = 0.0030, *P* = 0.0000 and *P =* 0.0000 respectively) and CORT groups (*P* = 0.0210, *P* = 0.0010 and *P =* 0.0333 respectively) were significantly upregulated compared with those in the OVA group. The HDAC9 expression in the H-YGW and M-YGW group, but not in the CORT group, was much higher than that in the OVA group (*P* < 0.0010, *P* = 0.0152 and *P* = 0.8955, respectively). The expressions of HDAC7 and HDAC9–11 showed dose-dependent responses to YGW. The expressions of HDAC1–6 and HDAC8 are shown in Table 2. There were no significant differences in the expressions of these proteins between the experimental groups (all *P* > 0.05).

**Table 2:** **HDAC1–6 and HDAC8 protein expressions in lung tissues after adoptive transfer**

**Proteins**	***You-Gui-Wan***			**CORT**	**OVA**
	**High (n = 5)**	**Moderate (n = 5)**	**Low (n = 5)**	**(n = 5)**	**(n = 5)**
HDAC1	0.90 ± 0.42	1.51 ± 1.02	0.84 ± 0.32	1.12 ± 0.89	0.45 ± 0.27
HDAC2	0.89 ± 0.41	1.16 ± 0.47	0.97 ± 0.52	1.14 ± 0.45	0.42 ± 0.27
HDAC3	0.73 ± 0.32	0.78 ± 0.35	0.88 ± 0.43	0.72 ± 0.42	0.37 ± 0.23
HDAC4	0.41 ± 0.20	0.77 ± 0.41	0.65 ± 0.24	0.59 ± 0.17	0.31 ± 0.22
HDAC5	0.53 ± 0.22	0.61 ± 0.25	0.60 ± 0.23	0.52 ± 0.19	0.28 ± 0.09
HDAC6	0.68 ± 0.16	1.01 ± 0.31^*^	0.77 ± 0.11	0.75 ± 0.36	0.41 ± 0.13
HDAC8	0.69 ± 0.15	0.81 ± 0.49	0.69 ± 0.37	0.61 ± 0.41	0.28 ± 0.19

^*^ *P* <0.05, compared with OVA.

### Lung histopathology and AEII

**Figure 6:**
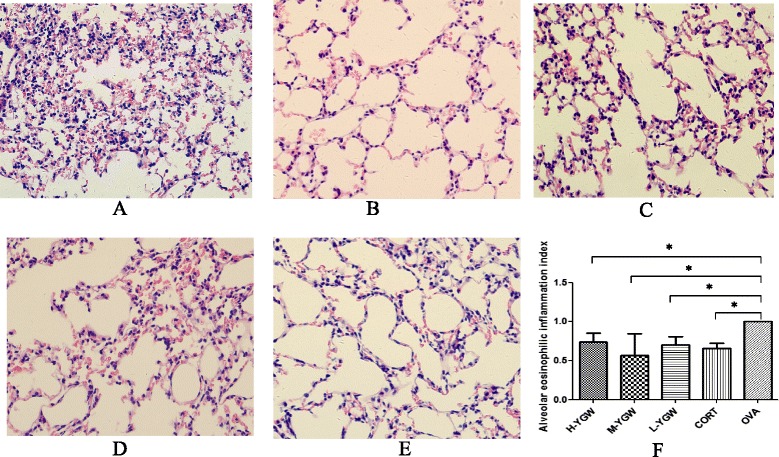
Pathological changes inthe lung tissues in the five groups of asthmatic mice after adoptive transfer (400×). **(A)** OVA group. **(B)** H-YGW group. **(C)** M-YGW group. (**D**) L-YGW group. **(E)** CORT group. **(F)** Alveolar eosinophilic inflammation index. ^*^ *P* <0.05, compared with OVA. Student-Newman-Keuls (SNK) test was used for multiple group comparisons.

The lung tissues in the OVA group were characterized by wider alveolar interstitium, congestion, and edema, with many infiltrating eosinophils and mononuclear cells including lymphocytes (Figure 6A). Compared with the OVA group, inflammation was reduced in the lungs of the H-YGW (Figure 6B), M-YGW (Figure 6C), L-YGW (Figure 6D), and CORT (Figure 6E) groups. These findings were reflected in the AEII values (Figure 6F): H-YGW (0.74 ± 0.19, *P* = 0.0146), M-YGW (0.57 ± 0.39, *P* = 0.0207), L-YGW (0.70 ± 0.18, *P* = 0.0069), CORT (0.66 ± 0.11, *P* = 0.0010) compared with OVA (1.02 ± 0.14). There were no differences in the AEII values among the H-YGW, M-YGW, L-YGW, and CORT groups (all *P* > 0.05).

### Relationships between cytokines, HDAC expression, and AEII

There were negative associations between HDAC activity and IL-4 (*rho* = −0.65, *P* = 0.009), IL-5 (*rho* = −0.59, *P* = 0.022), and IL-13 (*rho* = −0.65, *P* = 0.009) in BALF. There was a positive correlation between HAT activity and IL-13 in BALF (*rho* = 0.61, *P* = 0.015). These relationships between cytokines and HDAC or HAT activity also existed in cell cultures. Furthermore, the IFN-γ level in cell cultures was positively correlated with HDAC activity (*rho* = 0.59, *P* = 0.021). Significant correlations were found between IL-4 in BALF and HDAC5 (*rho* = −0.53, *P* = 0.0419), HDAC7 (*rho* = −0.68, *P* = 0.0055), and HDAC10 (*rho* = −0.61, *P* = 0.0167), IL-5 in BALF and HDAC11 (*rho* = −0.51, *P* = 0.0501), IL-13 in BALF and HDAC9–11 (*rho* = −0.62, *P* = 0.0141; *rho* = −0.51, *P* = 0.0519 and *rho* = −0.71, *P* = 0.0031 respectively), and IFN-γ and HDAC9 (*rho* = 0.56, *P* = 0.0308).

There were no correlations between T-bet/GATA-3 and IL-4, IL-5, IL-13, and IFN-γ. However, the GATA-3 protein level was negatively associated with HDAC activity (*rho* = −0.51, *P* = 0.05), HDAC8 (*rho* = −0.61, *P* = 0.002), HDAC9 (*rho* = −0.56, *P* = 0.004), and HDAC11 (*rho* = −0.60, *P* = 0.002). T-bet expression was negatively associated with HAT activity (*rho* = −0.58, *P* = 0.023), but positively associated with HDAC1–4 (*rho* = 0.52, *P* = 0.0071;*rho* = 0.59, *P* = 0.0017; *rho* = 0.43, *P* = 0.0330 and *rho* = 0.65, *P* = 0.0004 respectively), HDAC7 (*rho* = 0.44, *P* = 0.0293, and HDAC8 (*rho* = 0.63, *P* = 0.0008) .

HDAC activity was negatively associated with AEII (*P* = 0.05), but positively associated with HAT activity (*P* = 0.021). HDAC2–4 (*rho* = −0.50, *P* = 0.0361; *rho* = −0.61, *P* = 0.0201 and *rho* = −0.53, *P* = 0.0502 respectively) and HDAC10 (*rho* = −0.63, *P* = 0.0150) were negatively associated with AEII .

## Discussion

YGW, which is similar to CORT, dose-dependently increased HDAC activity, but reduced HAT activity in the lung tissues of asthmatic mice, and alleviated alveolar eosinophilic inflammation in their lungs by reducing GATA-3, IL-4, IL-5, and IL-13, and inducing IFN-γ release. Moreover, YGW reduced the production of inflammatory cytokines such as IL-4, IL-5, and IL-13 by increasing the corresponding activity of HDAC7/10, HDAC11, and HDAC9–11, respectively. YGW increased IFN-γ release by increasing HDAC9. Therefore, YGW appears to promote HDAC7- and HDAC9–11-induced histone deacetylation of Tm, leading toprotection against lung inflammation and eosinophilic infiltration.

Elevated levels of serum IgE are correlated with the incidence or severity of asthma. Undetectable serum total IgE may serve as a marker for immune dysregulation and autoimmunity [22]. Lin *et al.* [15] revealed that YGW treatment significantly decreased serum total IgE and possessed anti-inflammatory effects including reductions in total cell numbers and in the percentages of macrophages and eosinophils in BALF of *Dermatophagoides pteronyssinus*-induced asthma. Blood and tissue eosinophilia are hallmarks of allergic rhinitis, atopic dermatitis, and atopic asthma. Eosinophils are key effector cells in the pathogenesis of allergic disease [23] and are recruited from the circulation to inflammatory tissues in response to allergic stimuli [24]. In the present study, we chose the AEII as an index of inflammation, and found that YGW could reduce lung inflammation and eosinophil infiltration into the lung tissues to improve the asthmatic condition.

T cells with a Th2-like phenotype are involved in orchestrating the asthmatic inflammatory responses [25]. Lin *et al.* [18] found that YGW reduced the number of CD4^+^CD25^+^ T cells in BALF, suggesting that YGW might block this feedback system and shift Th2-dominance by increasing IL-12 levels. Notably, the important regulators of eosinophil trafficking are Th2 cytokines such as IL-4, IL-5, and IL-13 [26]. In our study, we demonstrated that YGW alleviated alveolar eosinophilic inflammation by reducing GATA-3, IL-4, IL-5, and IL-13, and inducing IFN-γ release. Suppression of these cytokines might reduce eosinophil infiltration into the alveolar spaces, thereby alleviating allergic asthmatic inflammation.

Su *et al.* [12] revealed that endogenous HDAC activity was involved in maintaining the balance of pre-established Th1-like and Th2-like responses, and inhibiting excessive Th2 immunity. In our study, we found that YGW dose-dependently increased HDAC activity and reduced HAT activity in the lung tissues of asthmatic mice, consistent with the results of Haczku *et al.* [27], thus supporting a role for the Th2-derived cytokines IL-4 and IL-5 in the induction of eosinophilic inflammation in our model. Therefore, we propose that YGW treatment reduced lung inflammation by downregulating Th2 cytokines. In addition, we found clear negative associations between HDAC expressions and the levels of IL-5 in cell cultures and BALF. IL-5 induces selective eosinophil (as opposed to neutrophil) recruitment [28] and activation [29], and is the only known cytokine to promote the terminal differentiation of eosinophil precursors and enhance survival [30].

The metal-dependent HDAC enzymes are grouped into class I, class II, and class IV on the basis of their homology to yeast proteins. Class I is comprised of HDAC1, HDAC2, HDAC3, and HDAC8, which are predominantly localized in the nucleus, have a ubiquitous tissue distribution, and play critical roles in cell survival and proliferation [31]. Class II enzymes are further subdivided into class IIa (HDAC4, HDAC5, HDAC7, and HDAC9) and class IIb (HDAC6 and HDAC10) [32]. Little is known about the function of HDAC10. Similarly, not much is known about HDAC11, the sole member of the class IV enzymes [33]. There is evidence that the different HDACs target different patterns of acetylation, and therefore regulate different types of genes [34]. In biopsies from patients with asthma, there was an increase in HAT activity and a reduction in HDAC activity, thereby favoring increased inflammatory gene expressions [35]. In our study, YGW reduced the inflammatory cytokines IL-4, IL-5, and IL-13 by increasing the activity of HDAC7/10, HDAC11, and HDAC9–11, respectively.

The findings that endogenous HDAC activity markedly influenced both the intensity and nature of immune responses suggest a potential mechanism through which treatment can alter the balance of Th1/Th2 recall responses by modulating endogenous HDAC activity [26]. Reactive oxygen species from cigarette smoke also reduce HDAC2 expression and activity, enhance cytokine expression, and inhibit glucocorticoid action in alveolar macrophages [36]. In severe asthma and asthmatics who smoke, HDAC2 is reduced, thus preventing corticosteroids from suppressing inflammation [37]. It was suggested that HDAC2 may be a novel target for the development of new anti-inflammatory treatments [37]. In our study, there were no differences in HDAC2 activity between the experimental groups, probably because our asthma was not severe and did not feature neutrophilic inflammation [38]. However, YGW did promote histone deacetylation of Tm by HDAC7 and HDAC9–11, leading to protection against eosinophil infiltration and lung inflammation.

GATA-3 is essential for T-cell development and the induction of Th2 cytokines (IL-4, IL-5, and IL-13). It is also an important transcription factor in the pathogenesis of asthma [39]. Ectopic expression of GATA-3 increased the expression of Th2-associated cytokines and decreased the expression of Th1-associated cytokines [40]. HDAC9-deficient mice exhibit increased Th2 polarization, with HDAC9 deficiency being associated with global site-specific lysine histone acetylation at H3 (H3K9, H3K14, and H3K18) that was localized to the IL-4 promoter [41]. In addition, HDAC9 has effects on Foxp3 expression and function (regulatory T cells) that suppress the production of Th2 cytokines [42]. Han *et al.* [43] found that transcriptional activation of the IL-5 gene by trichostatin A was achieved by affecting HDAC function on the IL-5promoter via transcription factors. Salidroside, a synthetic compound originally from Chinese herbal medicines, improved the progression of asthma, and could be used as a therapy for patients with allergic asthma by regulating the GATA-3/T-bet balance [44]. In our study, we did not find any correlations between T-bet and GATA-3 and their corresponding cytokines.

Glucocorticoid suppression of inflammatory genes requires, at least in part, recruitment of HDACs to the transcriptional activation complex by the glucocorticoid receptor [45]. We found that YGW had similar effects to CORT, with a dose-dependent increase in HDAC activity and reduction in HAT activity in an asthmatic mouse model.

However, airway hyper-responsiveness or IgE levels, which are valuable in the diagnosis of asthma and assessment of its severity, were not measured in the mouse model in this study.

## Conclusion

Histone deacetylation of Tm was observed during alleviation of asthma by YGW.

## Abbreviations

AEII: Alveolar eosinophilic inflammation index
BALF: Bronchoalveolar lavage fluid
CORT: Hydrocortisone
HAT: Histone acetyltransferase
HDAC: Histone deacetylase
IFN: Interferon
IL: Interleukin
OVA: Ovalbumin
Th1: T helper 1
Th2: T helper 2
Tm: Memory T lymphocytes
YGW: *You-Gui-Wan*
